# Supplementary material for: A Novel Hydroxyapatite/Vitamin B12 Nanoformula for Treatment of Bone Damage: Preparation, Characterization, and Anti-Arthritic, Anti-Inflammatory, and Antioxidant Activities in Chemically Induced Arthritic Rats
Source: Pharmaceuticals (Basel). 2023 Apr 6;16(4):551. doi: 10.3390/ph16040551 (PMC10143295; doi:10.3390/ph16040551)
Supplement: Supplementary file 1 [file pharmaceuticals-16-00551-s001.zip › pharmaceuticals-2200918-supplementary.pdf]

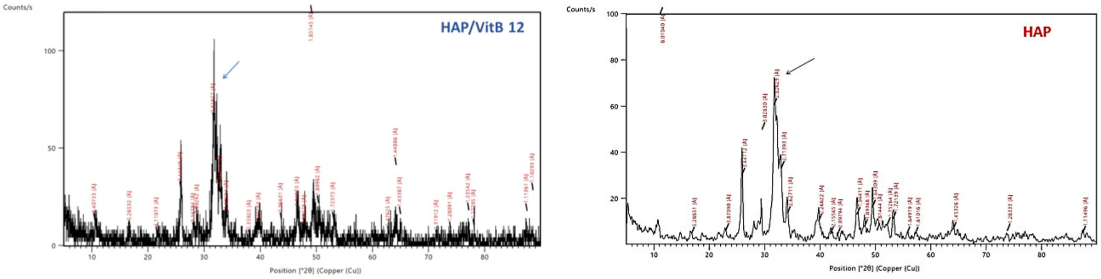

Figure. S1. The XRD of HAP and HAP/Vit B_{12} with showing the displacing of each band

Table S1 BJH Pore distribution adsorption results for HAP

BJH Pore Size Distribution Adsorption results

**Surface Area**	67.1843 m²/g
**Pore Volume**	0.489181 cc/g
**Pore radius Dv(r)**	1.92417 nm

Table - BJH Pore Size Distribution Adsorption

**radius nm**	**Pore Volume cc/g**	**Pore Surf. Area m²/g**	**dV(r) cc/nm/g**	**dS(r) m²/nm/g**	**dV(log r) cc/g**	**dS(log r) m²/g**
1.92417	1.305624e-02	1.357074e+01	2.597824e-02	2.700196e+01	1.144412e-01	1.189510e+02
2.58814	3.101684e-02	2.744988e+01	2.176111e-02	1.681600e+01	1.285770e-01	9.935851e+01
3.74849	5.326262e-02	3.931907e+01	1.487667e-02	7.937412e+00	1.266826e-01	6.759121e+01
6.57573	1.003017e-01	5.362596e+01	1.130987e-02	3.439885e+00	1.653750e-01	5.029863e+01
57.3639	4.891806e-01	6.718428e+01	3.991893e-03	1.391780e-01	3.573199e-01	1.245801e+01

Table S2 BJH Pore distribution adsorption results for HAP/Vit B_{12}

BJH Pore Size Distribution Adsorption results

**Surface Area**	55.8163 m²/g
**Pore Volume**	0.476947 cc/g
**Pore radius Dv(r)**	1.92195 nm

Table - BJH Pore Size Distribution Adsorption

**radius nm**	**Pore Volume cc/g**	**Pore Surf. Area m²/g**	**dV(r) cc/nm/g**	**dS(r) m²/nm/g**	**dV(log r) cc/g**	**dS(log r) m²/g**
1.92195	8.982582e-03	9.347375e+00	1.731701e-02	1.802027e+01	7.616805e-02	7.926133e+01
2.57439	2.027900e-02	1.812338e+01	1.436900e-02	1.116304e+01	8.450968e-02	6.565420e+01
3.72667	3.856419e-02	2.793654e+01	1.204247e-02	6.462865e+00	1.018903e-01	5.468176e+01
6.46664	8.010355e-02	4.078382e+01	1.048564e-02	3.242995e+00	1.511194e-01	4.673819e+01
52.7981	4.769469e-01	5.581632e+01	4.473927e-03	1.694731e-01	3.741287e-01	1.417205e+01
